# Vaccination strategies against COVID-19 and the diffusion of anti-vaccination views

**DOI:** 10.1038/s41598-021-85555-1

**Published:** 2021-03-23

**Authors:** Rafael Prieto Curiel, Humberto González Ramírez

**Affiliations:** 1 Centre for Advanced Spatial Analysis, University College London, Gower Street, London, WC1E 6BT UK; 2 University Gustave Eiffel, LICIT, 69518 Lyon, France

**Keywords:** Applied mathematics, Risk factors

## Abstract

Misinformation is usually adjusted to fit distinct narratives and propagates rapidly through social networks. False beliefs, once adopted, are rarely corrected. Amidst the COVID-19 crisis, pandemic-deniers and people who oppose wearing face masks or quarantine have already been a substantial aspect of the development of the pandemic. With the vaccine for COVID-19, different anti-vaccine narratives are being created and are probably being adopted by large population groups with critical consequences. Assuming full adherence to vaccine administration, we use a diffusion model to analyse epidemic spreading and the impact of different vaccination strategies, measured with the average years of life lost, in three network topologies (a proximity, a scale-free and a small-world network). Then, using a similar diffusion model, we consider the spread of anti-vaccine views in the network, which are adopted based on a persuasiveness parameter of anti-vaccine views. Results show that even if anti-vaccine narratives have a small persuasiveness, a large part of the population will be rapidly exposed to them. Assuming that all individuals are equally likely to adopt anti-vaccine views after being exposed, more central nodes in the network, which are more exposed to these views, are more likely to adopt them. Comparing years of life lost, anti-vaccine views could have a significant cost not only on those who share them, since the core social benefits of a limited vaccination strategy (reduction of susceptible hosts, network disruptions and slowing the spread of the disease) are substantially shortened.

## Introduction

Major disruptions have been suffered at all social levels due to the COVID-19 pandemic. With over 10,000 daily confirmed casualties directly related to the virus during December 2020, and an increase of deaths related to other illnesses due to weakened healthcare systems^1^, it will be among one of the leading causes of death of 2020 and it will have long-lasting economic and social consequences. Surveys conducted in heavily affected cities suggest that a small part of the population has acquired COVID-19 antibodies^2^, implying that we are far from reaching herd immunity through natural infection^3^. Furthermore, considering a conservative infection fatality ratio and an optimistic herd immunity threshold, several thousand casualties would still be suffered^3^. With many vaccines already approved, the biggest and most ambitious vaccination campaign in history has already begun.

The production and mass distribution of a COVID-19 vaccine will still be a major challenge. For example, Robert Redfield, the Director of the US Centers for Disease Control and Prevention (CDC), mentioned on a US Senate hearing (September 16th, 2020) that he expects that with the availability of the vaccine the US public will return to their “regular life” around the third quarter 2021^4^. In most parts of the world, mass immunisation will not happen any time soon, and therefore, a targeted vaccination will be a key element to optimise the limited number of vaccines and to mitigate the impacts of the pandemic. Under this scenario, unvaccinated individuals will still benefit from the immunity of those vaccinated, since the reduction of susceptible hosts limits the spread of the disease^5^. Considering the global demand for the vaccine, distinct vaccination strategies, prioritising population groups with higher exposure to the virus or higher risk of severe disease or death, will be a key policy, particularly during the early stages of the vaccine distribution.

Beyond the limitations related to the logistics of the vaccine, opposition to vaccination will become central to any strategy for coping with the pandemic. Already for COVID-19, the use of a non-invasive item such as the mask has been highly polarising and rejected by some groups, and protests and demonstrations around the world against the responses to the pandemic are frequent. Anti-lockdown protesters and conspiracy theorists have already demonstrated against the possibility of a mandatory vaccine, as is the case with pandemic sceptics and so-called “anti-vaxxers” in London^6^, whilst some conspiracy theorists burned 5G towers in the United Kingdom^7^. A survey conducted in 27 countries indicates that 1 in 4 people would not get a COVID-19 vaccine due to concerns about side effects and doubts about its effectiveness^8^, and another survey suggests that around half of the population would rather let others receive the vaccine first^9^, which could indicate both fears and solidarity with others. Also, some people fear more a mandatory vaccine for COVID-19 than the virus itself^10^. Vaccine opposition views have already reduced vaccination coverage in some places, causing the re-emergence of previously controlled diseases^11^, as happened for measles in 2019^12^. Anti-vaccination views offer a wide range of narratives, such as safety concerns, conspiracy theories and the use of alternative medicine, and it has been predicted that these views could dominate in a decade^13^. Different narratives about the virus itself^14^ and against the vaccine would friction vaccination strategies, which could greatly influence their coverage and the time needed to eliminate transmission^15^.

Either because of logistic restrictions or because people refuse to be vaccinated, strategies in which some people—perhaps a small percentage—are immunised, need to be considered in order to reduce the impacts of the pandemic. Here, we consider a network-based population model and five vaccination strategies varying the proportion of the population that has received the vaccine. A modified version of the SIR model, SVIR (due to the existence of the Vaccine) is used to detect the long-term behaviour of a pandemic, where the efficiency of each vaccination strategy is measured using the years of life lost and the time to eliminate transmission. Simulating the evolution of the pandemic under different network topologies allows us to compare vaccination strategies and detect successful immunisation strategies. Using a similar technique, we consider the dispersion of anti-vaccination views on a network, which pass through individuals with a certain level of *persuasiveness*. Assuming that all individuals are equally likely to adopt anti-vaccination views after being exposed to them, we demonstrate that individuals who are central in the network are much more likely to adopt these views. Considering that individuals who share anti-vaccination views will refuse a vaccine, we quantify the burden that anti-vaccination narratives have on optimal vaccination strategies.

## Modelling vaccination and anti-vaccination views on a network

### Dealing with non-universal vaccination

Vaccinations changed our health and quality of life dramatically. Yearly cases of measles, mumps, or smallpox decreased by more than 98% since introducing a vaccine^5^. Formerly fearsome diseases are now rare in many parts of the world thanks to vaccination programs^11^. Yet, due to a limited number of vaccines, their cost, views against vaccines and many more reasons, including the fact that with more vaccines, the spreading of a virus slows down, which in turn, tends to discourage more people from participating in the vaccination campaign^16^, universal vaccination is almost impossible. Thus, understanding the impact of limited vaccination is critical, as it plays a relevant role during the COVID-19 pandemic.

Vaccination strategies have two core objectives: lower and delay the peak size (“flattening the curve”) and reduce the final infected population to limit morbidity^17^. There are, however, many challenges for designing a vaccination strategy. Deaths can be prevented by first targeting highly vulnerable populations^3^ (such as elderly people, with severe morbidity, or vulnerable communities, including prison and homeless populations^18^). Yet, targeting vulnerable populations might not reduce the viral circulation and might not reduce the final infected population, particularly if the targeted population has limited social contacts. Another strategy is to target highly exposed people, such as health-care workers or with frequent social contacts, as it could slow down the overall exposure to the virus. Other strategies, for instance, targeting young people (as more years of life are saved with each vaccine) or random individuals (as means of some distributive fairness) could also be proposed for a variety of reasons. Thus, the relevance of being able to compare the expected outcomes of different vaccination strategies.

### Modelling immunisation in a population

One of the first mathematical models in epidemiology was concerned with immunisation. In 1760, Daniel Bernoulli predicted the impact of immunisation with cowpox upon the expectation of life of the immunised population^19^. Since then, many models of immunisation have been constructed. Remarkably, the Susceptible, Infected and Recovered (SIR) compartments model proposed in 1927 by William Ogilvy Kermack and Anderson Gray McKendrick^20^ has been extended for the application of a vaccine (SVIR)^17,21–24^.

Nearly 100 years ago, the lack of computational power meant that dealing with a high number of variables was nearly impossible and obtaining numerical results was very costly and so compartmental models in epidemiology were translated, under many assumptions, into differential equations. Algebraic solutions were more accessible than numerical results and gave rudimentary insights into the general behaviour of the dynamics of the disease^25^. For instance, this basic model can provide us with a rough idea of the number of individuals that would need to be infected to achieve herd immunity^18^. In the case of a vaccine, it was showed that uniform vaccination is always less effective than targeted vaccinations, and the optimal strategy involves vaccinating specific individuals first^26^.

Many of the implications of the SIR and the SVIR models rely on simplifying assumptions, such as an homogeneous population mixing and uniform recovery rate. In recent years, thanks to the gain of computational power and the development of specific tools and frameworks, some of these assumptions have been relaxed, particularly assuming an homogeneous population with homogeneous contacts. The use of networks in epidemiology is a powerful tool to relax both as tagged nodes allow considering disease dynamics for heterogeneous population groups^27^, and different distribution of the edges allows considering distinct types of contacts. For example, in the case of a sexually transmitted infection, the sexual partnership network is a natural framework for modelling the disease^28^. The contact network among people is frequently modelled as a strongly connected small-world-like graph^29,30^, meaning that the path in the network needed to connect any two nodes is rather small, or with many short-cut connections^31^, with a well-defined scale for the degree distribution of the number of contacts of each node^32^. The network structure has a major impact on the spread of infectious diseases and therefore, on successful vaccination strategies^5,28,33,34^. The basis of the disease models on a network is a compartments model (as with the SIR and SVIR model), but minor changes on the network’s connectivity might alter results significantly. Furthermore, the network structure appropriate for a given setting not only depends on the structure of the contacts of the population (which likely changes over time) but on the infection itself^28^.

Designing successful immunisation strategies need to consider the inhomogeneous connectivity properties of the network^35^. An infected person, even with a reduced number of contacts, can pass the virus between separate clusters, particularly if any of such contacts is a short-cut connection in the network^32^. On a small-world network, all individuals, even if they have a small number of contacts, are within a few infections to be infected themselves. On a different network structure, some nodes (often called “hubs”) have many more connections than others, and the network as a whole has a power-law degree distribution, as in the case of the network of sexual contacts, which exhibits scale-free features^35^, referred to as a scale-free network^36^. On a scale-free network, uniform vaccination is always less effective than targeted vaccination^28^. Scale-free networks are resilient to a few disconnections but are strongly affected by selective node damage. If a few of the most connected nodes are removed, the infection suffers a substantial reduction in its ability to propagate^35^. Control programs should be targeted towards the highly connected nodes, and such programs will be much more effective than those that target nodes at random^37^.

These strategies, however, assume that all individuals would accept the vaccine if offered. A targeted vaccination towards the most connected nodes will be highly ineffective if, for any reason, some of the central nodes refuse a vaccine. The impact of those who reject a treatment has been analysed among distinct demographic groups^15^, showing that non-adherence to a treatment, such as a vaccine, can greatly influence the needed coverage to eliminate transmission. In the case of network structures, the impact of non-adherence has not been explored, and it is likely to play a significant role during the COVID-19 vaccination program.

### Opinion dynamics and anti-vaccination views

An idea or an opinion, such as views in favour or against a vaccine, is transmitted—frequently as an intentional act—from one person to another^38^. Through distinct types of interactions, people want to persuade others to adopt an opinion^39^. The views of others might have an impact on individual beliefs^40^ who update their own opinion. Different ways to model, not only different perceptions or ideas but to also the updating process of those ideas—obtained through interactions with others or with some externalities—aim at capturing why opposing views (which could be the acceptance of a vaccine), can emerge and co-exist in a society, even if all individuals try to reach a consensus.

Opinion dynamics has been modelled through a variety of angles and techniques, for instance, kinetic models of opinion formation^41^, mean-field analysis, which usually leads to a system of differential equations^42^, agent-based models and even epidemiological models^38^. Frequently, two opposing opinions are assigned to the extremes of an interval, say, $[-1,1]$, individual opinions are modelled as a number *s* in that interval, according to the position with respect to the two opinions, and through interactions, individuals have some compromise (opinions get closer) and other elements, such as memory loss^43^, the presence of leaders^42^, the ability to convince others with a different opinion^44^, varying levels of assertiveness^45^, the fact that more extreme opinions are more difficult to change^46^. Also, confrontation with distinct views and opinions does not happen at random. Either because of the dynamic process through which opinions are updated^44^, or because of preferential interactions, people tend to be surrounded by others with similar views^45^.

As in the diffusion of a virus, different network structures are a natural framework to analyse opinion dynamics. Ideas and opinions tend to propagate on a network between adjacent nodes^47^. For instance, opinion leaders, who have a greater impact, can be the hubs of the social (scale-free) network^42^, whereas the distribution of online contents can pass through highly connected nodes on a (small-world) network^48^. The results observed of complex opinion dynamics are that a population might have relevant levels of polarisation or fragmentation^49^, people tend to have more interactions with others with similar views, so they are more frequently exposed to the information that aligns to their values (confirmation bias)^48^. Users tend to aggregate in communities of interest, which causes reinforcement and fosters confirmation bias, segregation, and polarisation and leads to the proliferation of biased narratives fomented by unsubstantiated rumours, mistrust, and paranoia^48^. Existent communities adopt new narratives and work as echo chambers, limiting the information their members receive to what they agree with and discarding contradicting points of view, thus reinforcing their beliefs. Moreover, repeated exposition to similar content increases the chances of believing it, even when its plausibility is low^50^. For example, it was detected that political ideology predicts perceptions of COVID-19 threat^51^.

Massive misinformation is becoming one of the main threats to our society^12,48^ and they have rapidly become central in terms of COVID-19^52^. Already for COVID-19, most of the misinformation detected involves reconfigurations, where existing (often true) facts are twisted or adjusted to fit different narratives^53^. With a vaccine for COVID-19, opposing views towards immunisation will become the crucial element, especially if the propagation of the virus itself and if vaccination strategies are, in any way, entangled within the network of individuals who refuse it.

### Anti-vaccination views

Opposition to vaccination is as old as vaccines themselves. The reasons behind anti-vaccine views are diverse, including religious^54^ and political beliefs^55^, as well as concerns about their safety and effectiveness, that are often based on misinformation. Perhaps, the most cited case in the last decades is the theory that falsely links autism in children with the MMR vaccine, a theory that has been debunked by several studies^56^, but that is still used as an argument against vaccination by some groups. The origin and motivation behind fake narratives are often unclear, but they usually create a certain panic in the readers and encourage action, such as sharing the “news” with family and friends^57^. Perceived altruism and solidarity with others is one of the main reasons people share unverified and often fake content^52,58^. Vaccine safety concerns receive more public attention than vaccination effectiveness^11^. People share false claims about COVID-19 and its vaccine since it is often difficult to detect whether some content is accurate^59^.

According to a 2018 study in 140 countries^60^, 79% of the global population perceive vaccines as safe, and 84% as effective. However, these percentages vary greatly from region to region, representing less than two-thirds of the population in some areas, especially Europe. And, in the case of a COVID-19 vaccine, a study published in August 2020 in 27 countries found that nearly one in four adults would not get a vaccine for COVID-19^8^ and in some countries, more than half of the population would not get it, including Poland and France. The main reasons cited for not taking the vaccine were adverse secondary effects and doubts about its effectiveness. Misinformation, amplified by social networks, have eroded the public confidence on vaccination, causing an increase in the number of outbreaks of diseases that were already controlled, as happened for measles in 2019^13^.

Currently, there are still some critical questions concerning the COVID-19 vaccines. Still, we do not know whether the immunisation of a vaccine would wane over time and so how long the acquired immunity would last^18^. Even with a vaccine, if reinfection could occur, persistent herd immunity may never be attained, which then could lead to cyclic outbreaks and cyclic vaccination^18,61^. Finally, we do not know if a sufficiently large population would reject the vaccine delaying even more the process of obtaining herd immunity.

## Methods

A population of *N* individuals is constructed with two attributes: current age (or simply age) and age at death (or simply death). Both age and death are sampled from a scaled Beta distribution: $100Beta(\alpha _a, \beta _a)$ with $\alpha _a =2$ and $\beta _a= 3$ for age, and $100Beta(\alpha _d, \beta _d)$ with $\alpha _d =5$ and $\beta _d= 2$ for age at the moment of death^62^. Individuals with death smaller than age are resampled, resulting in a simulated population with a median age of nearly 35 years and a median age at the moment of death of 75 years (close to the median age and expected life in Mexico City).

Three network topologies are considered, where the nodes represent the individuals and the edges their contacts, either concerning their vaccine views or their physical contacts, so they might pass the virus between them. Firstly, a proximity network, where nodes are located randomly on a square and pairs at a distance smaller than a certain threshold *d* are connected. The proximity network tends to have a large network diameter since nodes only contact others located nearby. Secondly, a small-world network with a rewiring probability *r*. The small-world network has shortcuts between the nodes, meaning that the network diameter is much smaller than the proximity network. Thirdly, a scale-free network with power *p*. The scale-free network has “hubs”, meaning some nodes with a much larger number of adjacent neighbours. The three networks have the same average node degree so that between different topologies, only the structure of the network is altered. Although very young or elderly people tend to have fewer contacts, we construct age and network independently, for simplicity.

An SVIR model is constructed in the network as follows. Firstly, some randomly-selected nodes are infected, and the rest of the nodes are susceptible. After the initial infection process, a percentage $\nu$ of the individuals are vaccinated, called the *vaccination rate*. If the individual is susceptible, she or he gains permanent immunity and is no longer capable of passing the virus, but if the person was already infected, the vaccine does nothing (a scenario which could happen for individuals who are asymptomatic and receive a vaccine). Each time step, which could be considered a day, susceptible individuals in contact with an infected person are also infected, with probability $\pi$. When a node is infected, it is moved to the recovered state after $\tau$ steps, counting from the moment of infection. A recovered node no longer passes the virus to others. The process stops when the population has no infected nodes, and that time is noted as *T*, i.e., *T* is the duration of the epidemic. See the Appendix for more details on the parameters of the model.

After infection, some of the individuals who “recover” might not survive. It has been noted that, in the case of COVID-19, lethality increases with age. For simplicity, we consider a linear impact of age, so that a person with age *y* years does not survive with a probability of $\phi y$, for some $\phi >0$. The average number of years of life lost due to the pandemic, denoted by *D*, is computed as the sum of the number of years that people who passed away lost (that is, their death minus their current age), divided by the population size *N*.

### Vaccination strategies

Five vaccination strategies, based on targeting people by their age or their position on the contact network, are considered. For the proportion $\nu$ of individuals receiving the vaccine:

- *Degree*—target the top $\nu$ nodes with a higher degree. More connected nodes receive the vaccine first.
- *Centrality*—vaccinate the top $\nu$ nodes with the highest node betweenness.
- *Peripheral*—apply the vaccine to nodes with the lowest node betweenness.
- *Age*—vaccinate the top $\nu$ nodes with highest age (elderly population) first.
- *Random*—select a portion $\nu$ of the individuals at random.

The first three strategies (Degree, Centrality and Peripheral) are based on network attributes, meaning that nodes are sorted depending on their degree or the node betweenness and the top $\nu$ nodes are vaccinated. The Age strategy is based on node attributes (their age), and the last strategy (Random) uses no information about the nodes.

Even though the vaccination strategies (in the case of the COVID-19) differ by country, they are mainly based on prioritising vulnerable groups, defined as those with significantly higher risk of severe disease or death, and those with high risk of contracting and transmitting the virus. The first group comprises elderly and adults with comorbidities; the second, health and education workers, as well as low-income persons living in dense urban neighbourhoods for which social distancing is difficult^63,64^. Three of the strategies that we test try to emulate the above vaccination guidelines. We identify the Age strategy (elderly first), with the group of higher risk of severe disease, and the Degree and Centrality strategies (prioritising people with many contacts or high betweenness centrality) with the group of population with a high risk of infection and transmission. It is worth mentioning that, in practice, individuals with a high degree can be identified by their activity (those with many contacts) but identifying those with high betweenness centrality is more complicated. For example, we could think of a university professor, who is in contact with several groups of students, or, at a larger scale, of individuals who make inter-city commute trips, thus acting as the bridge between different subnetworks. Finally, we define the Random and Peripheral strategies for comparison reasons since they are unlikely to be put into practice.

### Comparing vaccination strategies

We consider two distinct metrics for comparing vaccination strategies. Firstly, from the population of *N* individuals from which a small group is initially infected, vaccines are applied according to some strategy, and the SVIR dynamic is simulated until no individuals are infected. From the recovered individuals, the casualties are simulated and the average number of years of life lost *D* is computed as the sum of the difference between death and age from individuals who passed away, divided by the population size *N*. Although it is possible to simply count the number of casualties, measuring instead the average years of life lost distinguishes between the death of an elderly person, with a reduced number of years to live and the death of a young person. A second metric to understand the impact of distinct vaccination strategies is the time needed for the dynamic to stop, *T*, i.e., the time after the population has no infected nodes. Both *D* and *T* are computed for a vaccination strategy and for a certain vaccination rate $\nu$, so that $D_{Age}(\nu )$ and $T_{Age}(\nu )$ are reported for the Age strategy with a vaccination rate of $\nu$, and likewise for other strategies.

For a given network and a fixed vaccination rate and a (non-random) strategy, results might vary for two reasons. Firstly, because the initial infected population is sampled from the population. Secondly, because of the transmission of the virus itself: susceptible individuals who are adjacent to an infected node are infected with a probability $\pi$ on each time step, and so the progression of the virus might change between different realisations, even with the same initial conditions. For this reason, we simulate each vaccination strategy 500 times for different vaccination rates $\nu$ and report the intervals which contain $D_{S}(\nu )$ and $T_{S}(\nu )$ for the different strategies *S*.

Here, we consider a perfect vaccine that grants permanent immunity, although, in reality, the level of antibodies of an immune person may drop below a critical threshold (waning immunity), which could take individuals back into being susceptible^19,23^.

### Diffusion of anti-vaccine views

The diffusion process of anti-vaccine views plays a relevant role in the vaccination process and therefore, in the evolution and the burden of the pandemic. The social network is a crucial part of the diffusion process, as anti-vaccine views are spread through contacts, convincing other individuals of their views of the vaccine.

We construct a diffusion process on a network, similar to the SIR model, but for the adoption of an idea^38^ as follows. Initially, all individuals are susceptible to anti-vaccine views, and some randomly selected individuals have already these views. Although some individuals could be considered more susceptible (for instance, those who engage more with social media^52^), we assume that all individuals are equally susceptible. At each time step, individuals with anti-vaccine views share them with all their contacts. Individuals exposed for the first time to anti-vaccine views make a permanent decision based on the persuasiveness $\theta$ of the ideas, where $\theta \in [0,1]$ are the chances that an anti-vaccine view persuades the exposed individual. A small value of $\theta$ means that most individuals are not convinced by anti-vaccine views and the opposite for a high value of $\theta$. Since we assume that the decisions of people are permanent, then on the first step, only the neighbours of those who initially share anti-vaccine views are exposed to them, then, at each time step, only the neighbours of individuals who were convinced on the previous step are exposed for the first time to anti-vaccine views and make a decision. On the second step, only the neighbours of individuals who were convinced on the previous step are then exposed to anti-vaccine views, and so on. Each time step, only the neighbours of individuals who were convinced on the previous step are exposed for the first time to anti-vaccine views and make a permanent decision. The dynamic stops when no new individuals are convinced of anti-vaccine views.

At the final stage of this dynamic, some individuals have anti-vaccine views, some have been exposed to anti-vaccine views but do not support them, and some individuals (potentially a large group or none, depending on the persuasiveness of $\theta$) were never exposed to anti-vaccine views. We assume that only individuals who share anti-vaccine views reject a vaccine, and the rest, whether they were exposed or not to the views, will accept to be immunised if a vaccine is offered. A similar SIR model for the COVID-19 social media infodemic was constructed, although not based on a network, but based on the spreading parameters^65^.

## Results

**Figure 1:**
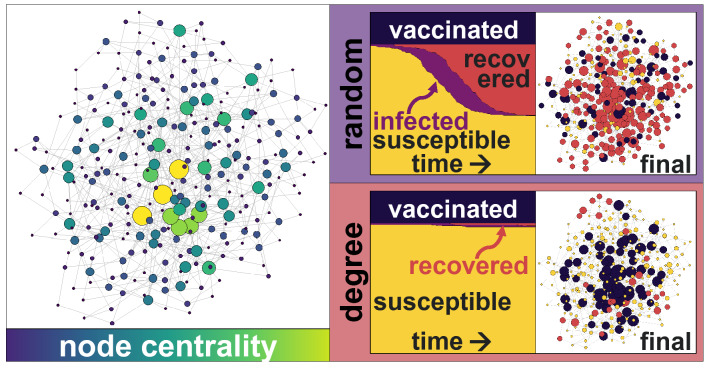
Schematic representation of a scale-free network with power $p=1$ (left) with larger and brighter nodes displaying their node betweenness. The evolution of the SVIR model for the Random and Degree vaccination strategies (centre), with the same vaccination rate $\nu = 20\%$, shows that the majority of the population will remain susceptible under the targeted Degree vaccination strategy, but that a large part of the population will be infected and recovered under the Random strategy. The final state of the nodes (right) shows that many nodes are recovered (red) with a Random strategy, but most remain susceptible (yellow) with a Degree strategy.

Considering a scale-free network with $N = 5000$ individuals and 250 infected individuals at time $t = 0$, targeted vaccinations yield drastically distinct results than a random vaccination strategy (Fig. 1). Applying the vaccine to central nodes not only protects them from receiving the virus, but also slows down its diffusion process, allowing nearby infected nodes to recover and stop passing the infection to others (as it has beenobserved previously^35,37^). The final size of the recovered population varies considerably. For example, considering a random vaccination strategy with a proportion of immunised people of $\nu = 20\%$, a large part of the population would be infected at a point. With targeted vaccinations, the size of the infected population drops drastically, and therefore most of the individuals remain susceptible, even with the same (small) vaccination rate $\nu = 20\%$.

**Figure 2:**
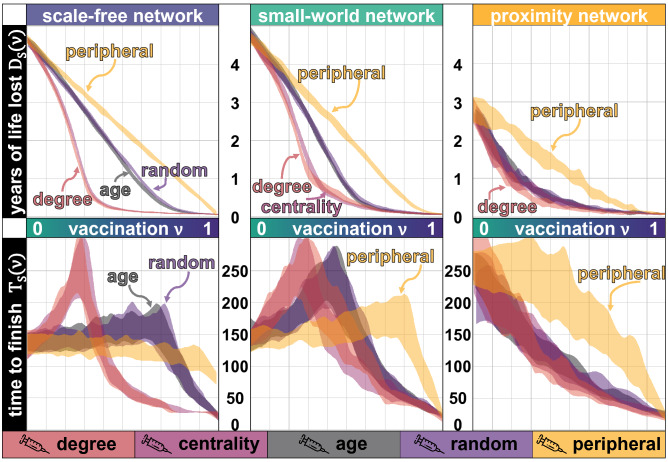
Years of life lost $D_{S}(\nu )$ (vertical axis) for different vaccination rates $\nu$ (in the top panel), and the corresponding time $T_{S}(\nu )$ (vertical axis) with the same vaccination rate (in the bottom panel). Three network topologies are tested, a scale-free network in the left (with power $p=1$), a small-world network in the middle (where the rewiring probability is $r=0.1$) and a proximity network in the right, all networks with an average node degree of 6. Each strategy is simulated 500 times with $N = 5000$ individuals, with a probability of infecting neighbouring nodes at each step of $\pi =0.05$ and with a varying vaccination rate $\nu$. The lethality of the virus increases with $\phi = 0.05$, meaning that a person with *y* years does not survive after being infected with a probability of 0.05*y*.

Without a vaccine, the simulations show that the population expects to suffer up to 4.7 years of life lost due to the pandemic. With a universal vaccine, the loss is negligible. Yet, a Random vaccination strategy with rate $\nu = 60\%$ has the same impact, in terms of life saved, than a Degree or Centrality strategy with $\nu = 30\%$. In a population in which some individuals are much more connected than others (a scale-free network), or one in which individuals are just a few nodes away from each other (a small-world network), targeting nodes with a high number of neighbours (Degree strategy) or nodes with a high betweenness (Centrality strategy) yields similar results in terms of the years of life saved (Fig. 2 top panel). On a proximity network, which has no nodes with a high number of neighbours or shortcuts, the life lost due to the pandemic is smaller as the virus propagates slower and among fewer nodes, nonetheless, a Degree strategy still reduces the burden of the pandemic, particularly if the vaccination rate is small (Fig. 2).

Surprisingly, targeting the most vulnerable population (in our model, elderly population, who are more likely to pass away after the infection) does not give better results in terms of the years of life lost than a random strategy, as it does not slow down the diffusion process of the virus as much as the Degree of the Centrality strategy. The Peripheral strategy gives the worst results in terms of the years of life saved, as it targets the nodes less capable of slowing down the virus’s diffusion or decreasing the number of deaths. The Age strategy does perform better than the Random strategy in terms of the number of casualties, but not in terms of the years of life lost. Measuring only casualties does not give sufficient information, and the demographics (age) of the population who passed away should be taken into account. The Age strategy targets individuals who are more likely to pass away, but it does not save much more years of life than the Random strategy, as it prevents casualties from people with a shorter remaining life expectancy, provided that the potential correlations between age and centrality are ignored. However, elderly people tend to have fewer contacts^3^, so targeting them with a low vaccination rate could imply some peripheral vaccination as well.

Vaccination, particularly with a low rate $\nu$, slows down the virus’s evolution, and thus it also slows down the time for the process to finish (Fig. 2, bottom panel). Without vaccination, it takes around 130 steps for the virus to spread across the population and for them to recover. Applying a vaccine to the most connected or the most central nodes (Degree and Central strategy) prevents the virus from spreading to many nodes, however, it still moves through the network between less central nodes, taking up to twice as many steps for the pandemic to end, compared to the case with no vaccine. The Age and the Random strategy also friction the evolution of the virus and, therefore, it also takes longer for the pandemic to end. With Degree, Centrality, Age and even the Random strategy, applying the vaccine to only some individuals (more than half of the individuals in some cases) delays the end of the pandemic. Only if a very large part of the population gets vaccinated, the pandemic finishes faster than without any vaccine.

The Peripheral strategy on a scale-free network accelerates the end of the pandemic (with all vaccination rates), since it immunises individuals who would be infected last, as opposed to the case of a small-world network. On a small-world network, less connected nodes still pass the virus to their neighbours. Therefore, low vaccination rates delay the epidemic process, increasing the duration of the pandemic $T_{S}(\nu )$.

Notice that although the SVIR model is a dynamic process, it is based on a static network of individuals, meaning that no new connections are formed. However, if the vaccine process takes longer, it is likely that new contacts (more edges) are added to the network, with its possible implications in terms of the years of life lost $D_{S}(\nu )$ and the time $T_{S}(\nu )$.

### The topology of the network

The topology of the social network plays a relevant role in detecting the optimal vaccination strategy and its impacts on saving lives and speeding the pandemic process. Targeting the most central or the most connected nodes on a scale-free network rapidly decreases the years of life lost for some vaccination rates, but it is slightly less effective on a small-world network and has a limited impact on a proximity network, compared to other strategies.

Qualitatively speaking, except for the Peripheral vaccination strategy, a small-world and a scale-free network show similar results. Targeting the vaccine to the most central or the most connected nodes reduces the casualties, although, for low vaccination rates, it increases the time for the pandemic to end. Targeting the most vulnerable individuals (even assuming that they are as central or connected as other individuals, which might not be true) has an impact as reduced as the Random vaccination strategy and, for low vaccination rates, both will increase the time $T_{S}(\nu )$. When central nodes are removed through vaccination, fewer individuals will be infected, but the virus takes longer to propagate, so the time $T_{S}(\nu )$ nearly doubles as compared to the scenario with no vaccination.

A strategy targeting the younger nodes could also be designed, but due to their small lethality, that strategy saves fewer years of life and prevents fewer casualties than any other strategy. The time $T_{S}(\nu )$ of such strategy follows the same patterns than the Age and the Random strategies since none of those strategies uses the network properties.

### Anti-vaccine views

**Figure 3:**
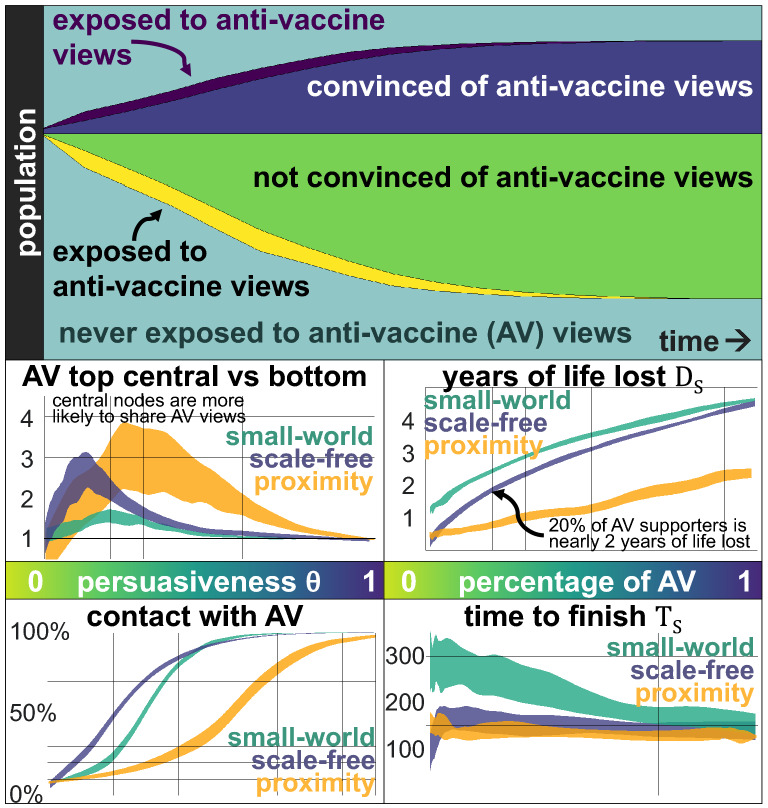
Anti-vaccine views are shared on a network of $N=5000$ individuals, where some randomly-selected nodes initially share the views. The top panel shows the evolution of those who are exposed for the first time to anti-vaccine views (dark purple) and are convinced by them (dark blue), those who are exposed for the first time (yellow) but do not adopt them (green) and individuals who never have contact with them (light blue). The odds of a person sharing anti-vaccine views, comparing the top 10% most central nodes against the bottom 10% (central left panel) shows that for extreme values of $\theta$, centrality does not have an impact (since all individuals share similar views), but for values in between, the most central nodes are two or more times more likely to share anti-vaccination views since they are more likely to be exposed. Since individuals are equally likely to adopt the views after their first exposure, more central individuals are more likely to adopt them. The impact is more pronounced on a proximity network (yellow) even when the network does not have nodes with a high degree, and a scale-free network (blue) than on a small-world network (green). By the end of the diffusion process of the anti-vaccination views (bottom left panel), even with a small persuasiveness $\theta$, most individuals will have contact with those views. On a scale-free network, with persuasiveness of $\theta = 0.3$, around 70% of the population has contact with the views (even if most of them reject them and do not pass them onwards). The impact of the size of the anti-vaccine community (AV) is relevant in terms of the years of life lost $D_{S}(\nu )$ (central right) and has some impact in terms of the time to finish $T_{S}(\nu )$, considering a vaccination rate of 30%. Assuming a Degree vaccination strategy, we see that if 20% of the people support anti-vaccination views, nearly two years of life are lost (so that $D_{S}(0.3) \approx 2$ years). However, with a vaccination rate of 0.3 and the Degree vaccination strategy, only 0.3 years of life are lost.

Anti-vaccine views strongly depend on the persuasiveness $\theta$. For small values of $\theta$, the idea dies fast, and only a few individuals ever share those views (Fig. 3). As with an infection, most of the individuals remain “susceptible”. For medium values of $\theta$, many individuals will have heard of anti-vaccination views, although many of them will not be convinced by them. Only with high values of $\theta$, anti-vaccination views percolate the network. The final size of the anti-vaccination community depends, in a non-linear way, on the persuasiveness $\theta$ and the network’s topology.

More central nodes are more exposed to anti-vaccination views than peripheral nodes (Fig. 3). In turn, assuming that nodes are equally likely to adopt anti-vaccination views the first time they are exposed to them, more central nodes are also more likely to share anti-vaccination opinions. The top 10% most central nodes are between two and three times more likely to share anti-vaccination views than the 10% least central nodes for a small persuasiveness $\theta$.

For small values of persuasiveness $\theta$, most individuals have contact with the anti-vaccination views, even if they reject them and do not pass them onwards (bottom left panel of Fig. 3). This can be particularly challenging, as anti-vaccine views have many narratives, including different conspiracy theories, safety concerns, the use of alternative medicine or medical risks such as autism^66^. Each narrative might follow similar dynamics, reaching most individuals but convincing only some, triggering collective narratives and creating echo chambers which reinforce themselves. Thus, the diffusion of distinct anti-vaccination narratives could also be conceived as many realisations of the same dynamic, each for separate views that convince different susceptible individuals.

The final size of the anti-vaccine community is directly related to the persuasiveness parameter $\theta$, and the number of steps needed for the dynamic to stop is surprisingly short. With a small persuasiveness $\theta$, the idea dies fast; with a large $\theta$, the idea percolates in just a few steps. Only with intermediate values of $\theta$, there is some delay, but it is still a fast process (around 10% of the steps needed for the virus dynamics). A virus might propagate fast, but fake news and misinformation propagate many times more quickly.

Finally, assuming that the network in which opinions are shared is the same in which the virus propagates, the anti-vaccination views’ impact is highly relevant and interferes with vaccination strategies. Considering a vaccination rate of 30% and a Degree vaccination strategy, 0.3 years of life are lost on a scale-free network and little more than a year on a small-world network, but that could increase to nearly two years if only 20% of the population shares anti-vaccination views (Fig. 3, right panels). More central individuals are the key aspect of optimal vaccination strategies, as they slow down the diffusion process of the virus, but their centrality also places them as nodes more likely to share anti-vaccination views, thus, having a substantial cost on the vaccination strategy. A larger anti-vaccination community reduces the time to finish $T_S$ as the virus propagates faster, except for a small range on a scale-free network only.

### Diffusion of anti-vaccination views and the virus on different networks

Opinions and anti-vaccine views are frequently shared on social media among distant individuals. Therefore, the network in which anti-vaccine views propagate does not match the network in which the virus infects more people, although there could be some interdependence. If a person rejects a vaccine, then the people with whom they have physical contact with are more likely to reject it as well, as they will probably be exposed to anti-vaccine views. Therefore, clusters of individuals will form who reject the vaccine and in which the virus spreads easily. To detect the impact of different network structures on the process of vaccination and the propagation of anti-vaccine views, first, we construct the opinion network according to some topology (proximity, small-world or scale-free). Then by dropping edges, rewiring them, or changing the topology completely, we construct the contact network in which the virus is passed.

**Figure 4:**
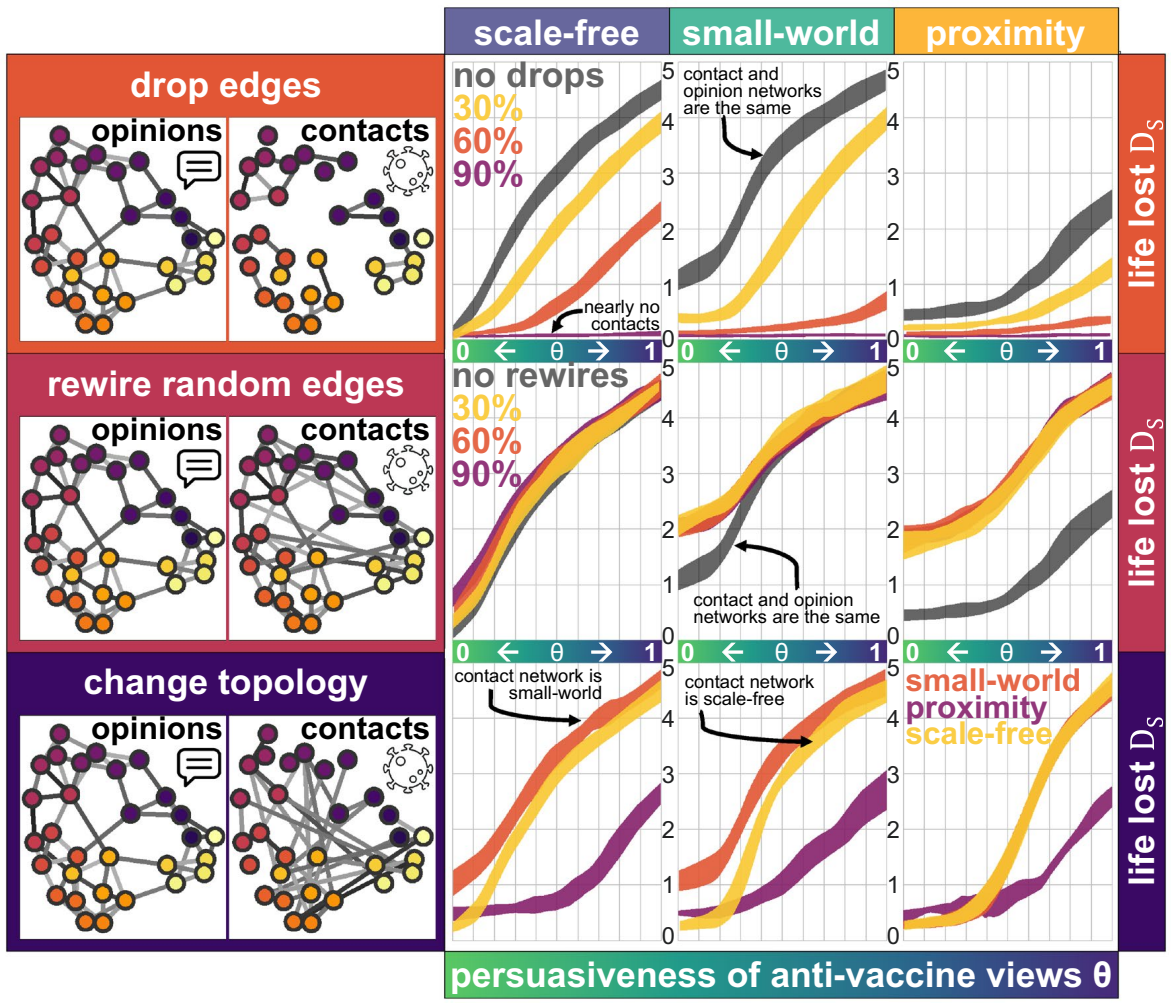
Anti-vaccine views are shared on a network of $N=5000$ individuals on a scale-free network (left), a small-world network (middle) and a proximity network (right), where some randomly-selected nodes initially are exposed to the views and share them according to the persuasiveness $\theta$ of the anti-vaccination views (horizontal axis). The contact network is constructed based on the opinion network by dropping edges (top panel), rewiring some edges (middle panel) or by changing the topology completely (bottom panel). The number of years of life lost at the end of the pandemic are measured, considering a vaccination rate of 30% and applying a Degree strategy, based on the degree of the contact network (vertical axis, $D_{S}(0.3)$).

Dropping some randomly selected edges from the opinion network to construct the contact network happens, for example, if two individuals are connected to each other through social media, but they do not have any physical contact and thus the edge between them should be dropped for the contagion process. Formally, the opinion network is constructed (as before, with some pre-determined topology and parameters) and then, each edge in the network is kept with a probability $\rho$ in the contact network. With $\rho = 1$, the contact network remains the same as the opinion network, with a value of $\rho = 0$ the contact network is empty, but with values of $\rho \in (0,1)$, the resulting contact network keeps some of the aspects of the opinion network, such as hubs, but with fewer edges. Results show that dropping random edges considerably decreases the speed at which the virus spreads in the network, thus reducing the final size of the recovered population and the life lost (Fig. 4, top panel).

Rewiring some randomly-selected edges of the opinion network to construct the contact network maintains some structure from the opinion network, but creates shortcuts between nodes^29^, increasing the burden of the pandemic (Fig. 4 middle panel). This could happen if, for example, two individuals have some physical contact (on the elevator or at the market), so that the virus can pass between them, but they do not interact in terms of their opinions or views concerning the vaccine. Formally, the opinion network is constructed as before, with some pre-determined topology and parameters. Then, each edge in the graph is maintained with a probability $\rho$, and it is rewired in any of the two extremes with a probability $1-\rho$ to a randomly selected node (ignoring loops and duplicated edges). For values of $\rho = 1$ the contact network is identical to the opinion network, but with smaller values of $\rho$, the contact network exhibits some perturbations. Even for values of $\rho \approx 0$ a node with a high degree is expected to keep a high degree after rewiring, meaning that part of the structure of the network is kept. Rewiring, even a small number of edges, creates contacts between nodes which are far away and increases the years of life lost. This is observed specially on the proximity network, in which clusters of anti-vaccine individuals get in touch with others who may not have exposure to such views, decreasing the collective benefit from the vaccination strategy. On a small-world network, only with a small persuasiveness $\theta$ of anti-vaccine views, rewiring creates contacts between anti-vaccine clusters and nodes that were not exposed, so there is a high cost on rewiring.

Considering that the propagation of the virus and the anti-vaccine views on distinct networks is the case when, for example, most of the debate regarding views in favour or against a vaccine happens online and rarely in person. Results show that the proximity network reduces the years of life lost when anti-vaccine views have a high level of persuasiveness, but that with a small level of persuasiveness, the scale-free network reduces the life loss since even a low vaccination rate minimises the propagation of the virus drastically (Fig. 4 bottom panel). If anti-vaccine views are seductive and they tend to propagate through a highly-connected network with hubs and shortcuts (almost all social media platforms) most people will be exposed to such views. If, in addition, there is a limited number of vaccines, applying them to the most central nodes which are not against the vaccine, will reduce the total burden of the pandemic. Further, preventing shortcuts in the contact network (such as people travelling over long distances) and hubs (people with too many contacts) lowers the number of years of life lost and the number of casualties, even with a limited number of vaccines and seductive anti-vaccination views.

## Discussion

A vaccine for COVID-19 will not be a silver bullet to end the pandemic and mitigate its impact. Beyond the logistics related to producing and distributing billions of vaccines worldwide, most countries will face limited availability of the vaccine, especially at early stages. Still, outbreaks can be contained by a strategy of targeted vaccination combined with early detection^32^.

Our simulations cannot be used to estimate the years of life lost we would experience due to COVID-19 for different vaccination rates since that requires considering comorbidities, access to health services and many more factors. Still, with a simulated population and pandemic, we obtain a qualitative description of how the process might evolve, considering a limited vaccination and anti-vaccination views.

The main benefit of a person being vaccinated does not rest just on the immunity that they gain, but also on the fact that they stop spreading the virus to others. As such, targeting vaccination to more exposed people or with a high centrality on the network (such as a person who works at a shop, a barber or a taxi driver, as they have frequent contact with many different clusters of people) reduces the final size of the recovered population and the expected years of life lost as well.

### Anti-vaccine views will be highly relevant

Anti-vaccine views and in general, fake news related to the virus are a second pandemic to defeat. Convincing individuals that a vaccine designed in a specific country (say the US or Russia, for instance) will feed conspiracy theories and other narratives, including its potential lack of testing and fast-track design. A vaccine for COVID-19 would raise many questions, and people often find miss-guiding, incomplete or entirely wrong answers to all of them on social media. Anti-vaccination narratives offer a wide range of attractive and seductive explanations which could pull undecided individuals, with genuine questions and concerns about the vaccine, into their anti-vaccination views.

Strictly enforced vaccination would blend perfectly with many of the conspiracy theories created around COVID-19 and would create massive chaos, considering the reactions against a non-invasive element, such as a face mask. If universal vaccination in a country is offered, still many individuals will likely opt to refuse it. In turn, the time needed to reach herd immunity might increase substantially.

Although pandemic-denying, opposing quarantines or face masks and anti-vaccine views are substantially different and are not necessarily shared by the same people, a pandemic-denier, for instance, will most likely oppose being vaccinated against COVID-19. A person with anti-vaccine views likely has as many contacts as she or he had before the pandemic and, without keeping some physical distance to others or adopting safety measures (such as a face mask), would still pass on the virus (to as many contacts) if they get infected.

One of the severe challenges with anti-vaccine views is that they might be shared by individuals with high centrality in the social network, as they are more exposed to them. Our results suggest that individuals with higher centrality are more likely to share anti-vaccination views, which is detrimental for the results of an optimal vaccination strategy. Central nodes play a highly relevant role in the diffusion of anti-vaccination views and the pandemic evolution.

Clear and concise evidence-based communication to the broad audience will be crucial to fighting against anti-vaccination views. Thus, it is worth considering an efficient communication campaign as having a similar impact as a vaccination strategy among the most central nodes. During the Ebola crisis, for example, religious leaders across faiths in Sierra Leone advocated for practices such as hand washing and safe burials and their engagement was considered a turning point in the epidemic response^67^. Social media celebrities, Instagram influencers or YouTube stars, could help promote evidence-based views about the vaccine but could also act as hubs of misinformation. An effective communication strategy, targeting central nodes with shreds of evidence about vaccines in general, and about COVID-19, could be viewed as an immunisation strategy against fake news which could percolate the network.

## Figures and Tables

**Figure 5:**
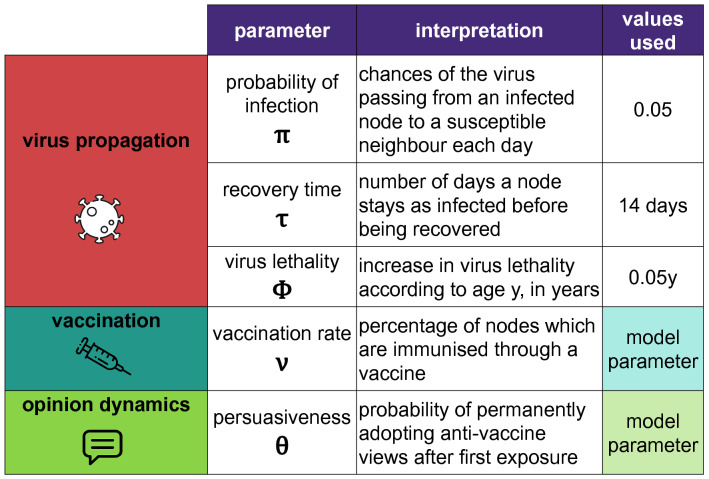
Main parameters used to model the propagation of some virus on a network, under some vaccination rate and strategy, where some individuals adopt anti-vaccination views. In terms of the virus, the three parameters are the probability of infection $\pi$, the recovery time $\tau$ and the lethality $\phi$ and are somehow related. The probability that a susceptible person is infected by an adjacent infected node is $1 - (1-\pi )^\tau \approx 0.51$ *(for the values of the parameters used)*since they have interactions for a period of $\tau$ days. The model parameters, which are used to detect the impact of anti-vaccine views and to measure the results of a vaccination strategy are the vaccination rate $\nu$ and the persuasiveness of the views $\theta$, which are considered for all of its potential values in the [${0, 1}$] interval.

## Data Availability

Code for simulating the SVIR dynamics and the propagation of anti-vaccine views, using^68,69^, is available at https://github.com/rafaelprietocuriel/vaccination.

## Appendix

### Parameter calibration

The models presented here for the diffusion of a virus and the propagation of anti-vaccine views are based on simplifying a complex reality. The diffusion of the virus and the way it is shared among infected individuals who recover after some days and the gain in popularity of anti-vaccination views are both severe social issues for which no model will ever capture all aspects at an individual and a collective level. Social models are frequently incomplete and inaccurate, aiming to capture emergent behaviours at a collective level, often reducing most elements of the process to its minimal expression. Here, the propagation of anti-vaccine views, for instance, is modelled as some permanent individual decision shared on some network. The propagation model depends on some assumptions, such as the fact that all anti-vaccine narratives can be grouped and modelled within the same dynamics, the fact that individuals make a permanent choice to adopt or reject the views or the fact that opinions are shared on a network between adjacent nodes. The main parameters of the model were selected to mimic, to an extent, the COVID-19 pandemic, including the propagation of the virus and the anti-vaccine views related to it, expecting to obtain a general description of both issues, but not hoping to obtain trends or forecasts concerning the pandemic (Fig. 5).

Other parameters, particularly concerning the three network topologies and the rewiring algorithm, were calibrated to keep the networks comparable in terms of their connectivity since a diffusion process would, in general, happen faster and have a larger recovered population on a network with higher connectivity. Thus, the topologies used help us test a network with a large diameter (the proximity network), the presence of shortcuts (the small-world network) and of hubs (the scale-free network) considering the case in which nodes have the same average number of neighbours.
